# Skåne Emergency Department Assessment of Patient Load (SEAL)—A Model to Estimate Crowding Based on Workload in Swedish Emergency Departments

**DOI:** 10.1371/journal.pone.0130020

**Published:** 2015-06-17

**Authors:** Jens Wretborn, Ardavan Khoshnood, Mattias Wieloch, Ulf Ekelund

**Affiliations:** 1 Department of Emergency Medicine, Skåne University Hospital, Lund, Sweden; 2 Department of Emergency Medicine, Skåne University Hospital, Malmö, Sweden; 3 Department of Clinical Sciences, Lund University, Lund, Sweden; University of Michigan, UNITED STATES

## Abstract

**Objectives:**

Emergency department (ED) crowding is an increasing problem in many countries. The purpose of this study was to develop a quantitative model that estimates the degree of crowding based on workload in Swedish EDs.

**Methods:**

At five different EDs, the head nurse and physician assessed the workload on a scale from 1 to 6 at randomized time points during a three week period in 2013. Based on these assessments, a regression model was created using data from the computerized patient log system to estimate the level of crowding based on workload. The final model was prospectively validated at the two EDs with the largest census.

**Results:**

Workload assessments and data on 14 variables in the patient log system were collected at 233 time points. The variables Patient hours, Occupancy, Time waiting for the physician and Fraction of high priority (acuity) patients all correlated significantly with the workload assessments. A regression model based on these four variables correlated well with the assessed workload in the initial dataset (r2 = 0.509, p < 0.001) and with the assessments in both EDs during validation (r2 = 0.641; p < 0.001 and r2 = 0.624; p < 0.001).

**Conclusions:**

It is possible to estimate the level of crowding based on workload in Swedish EDs using data from the patient log system. Our model may be applicable to EDs with different sizes and characteristics, and may be used for continuous monitoring of ED workload. Before widespread use, additional validation of the model is needed.

## Introduction

In recent years, there has been a steady rise in the number of emergency department (ED) patient visits in Sweden [1], and ED crowding is a growing problem in Sweden as well as internationally [2–4]. ED crowding has been associated with a decrease in quality of care and an increased risk of adverse outcomes for patients, including increased mortality rates [5]. However, in order to effectively estimate, prevent and minimize the negative effects of crowding, an objective definition is needed. Several models have been presented to define and objectively quantify ED crowding, the most noted including NEDOCS, EDWIN and ICMED [6–7]. Both NEDOCS and EDWIN were created in the United States. The NEDOCS model was made by correlating ED performance measures to staff assessments of crowding at academic centers, while EDWIN is based on ambulance diversion. Both models have been validated externally with mixed results [6]. The ICMED model was developed in the UK based on physicians perception of crowding and patient danger but has not yet been validated externally [7]. Ideally, crowding models should allow repeated or continuous estimation of the severity of crowding, and the immediate causes of it. Due to differences in the health care systems between Sweden and the United States neither NEDOCS or EDWIN is applicable to our EDs and some of the included measures in the ICMED model makes it difficult to measure automatically intra-operatively in the ED (ambulance offload time, patients left without being seen).

In the present study, we aimed to create an objective ED crowding model based on the EDs staff’s assessment of their workload, using data in the computerized ED patient log system.

## Methods

### Emergency Departments

The study was conducted at all 24-hour EDs in Region Skåne in southern Sweden: Skåne University Hospital at Lund and Malmö and the general hospitals in Helsingborg, Ystad and Kristianstad. These EDs use the same patient log system and are representative of a majority of the EDs in Sweden. The leadership at each ED consented to participation by their ED verbally and in writing. Characteristics of the included EDs are presented in Table 1. All EDs use the RETTS [8] system to prioritize patients based on their acuity, a system used in a majority of EDs in Sweden.

**Table 1:** ED characteristics.

Emergency Department	A	B	C	D	E
**Hospital**	Skåne University Hospital Lund	Skåne University Hospital Malmö	Helsingborg General Hospital	Ystad General Hospital	Kristianstad General Hospital
**Approx. number of hospital beds**	710	660	380	160	280
**ED beds**	35	72	38	16	24
**Approx. annual number of ED visits**	65000	85000	65000	30000	50000
**Admission rate**	30.3%	24.7%	24.1%	33.6%	30.5%
Trauma level*	1	1	1	2	3
**Spectrum of patients managed**	Internal Medicine, Neurology, Surgery, Orthopedics, Trauma, Infectious Diseases	Internal Medicine, Neurology, Surgery, Orthopedics, Trauma, Infectious Diseases	Internal Medicine, Neurology, Surgery, Orthopedics, Trauma, Infectious Diseases, Pediatrics	Internal Medicine, Neurology, Surgery, Orthopedics, Pediatrics, Gynecology	Internal Medicine, Neurology, Surgery, Trauma, Orthopedics, Infectious Diseases, Pediatrics, Gynecology
**Acuity**	High	High	High	Low	Low
**EM Specialist training program**	Yes	Yes	Yes	No	No

* trauma level according to the American College of Surgeons

### Data Collection

The head attending nurse and physician at each ED were asked to assess the ED workload at 250 separate time points (50 per ED) from March 11 to March 31, 2013. The time points were assigned by computer randomization with the following options: 04:00, 08:00, 12:00, 16:00, 20:00 and 24:00. Before randomization, the 04:00 and 24:00 time points were set to half the frequency of the other time points, since workload is generally lower during nighttime, and assessments are logistically harder to collect. Assessments were collected on paper questionnaires in 4 EDs and through a computer based questionnaire in 1 ED (Helsingborg). Each assessment was made by answering the question, “How would you assess the overall workload in the ED during the previous hour?” on a scale from 1 to 6, with 6 representing a very high level of workload. We assumed that a time window shorter than 1 hour would increase the variability in the assessments and in the patient log data, and that a longer time window would decrease the reliability of the assessments. All nurses and physicians were given written instructions when making the assessment but were not trained in assessing the workload prior to the data collection. An assessment was considered complete if both the head nurse and physician answered the question and partially complete if either answered it. The mean value of the head nurse and physician assessments at each time point was used in the analysis. In partially complete assessments, the available value was used in the analysis.

### Variables

Variables analyzed for possible inclusion in the model were identified based on the clinical experience of two senior attending emergency physicians and one senior attending nurse, as well as the published literature [6, 9]. From an initial set of 83 potential variables, we selected 14 variables, that were 1) accessible via the computerized ED patient log system, 2) considered relevant to the Swedish emergency care system, and 3) independent of the size of the ED and the hospital. Since there are no records of the number of ED personnel in the patient log system we assumed that the number of physicians and nurses registered as responsible for at least one patient during the previous hour would be a good estimate of the active ED staff numbers.

### Regression Model

Data were exported from the patient log system as Microsoft Excel files. We performed a multiple linear regression analysis comparing variables against the mean workload assessment at each given time point. Variables were analyzed for possible collinearity before inclusion in the regression model, and correlation between the individual variables and workload assessment was evaluated prior to the regression analysis. A model with 14 selected variables was postulated to provide the best estimate of the workload assessment and was denoted the full model. By regression analysis, we then filtered out variables with a p-value < 0.05 to create a reduced model that approximated the performance of the full model.

With the reduced model, we also retrospectively calculated the mean model score over the whole collection period at each ED to estimate possible differences in workload.

### Validation of the Model

After creating the reduced model, we prospectively collected two separate data sets at the two largest EDs to test the performance of the reduced model against workload assessments made by all nurses and physicians present in the ED, and to study the correlation between assessments by the head staff and the rest of the personnel. Workload assessments were collected during seven consecutive days at Lund (April 29 to May 5) and fourteen consecutive days at Malmö (July 22 to August 4). The same question and time points as above were used, except that 04:00 was omitted and 24:00 was replaced by 23:00 for practical reasons.

During the validation at Lund, assessments were made by every working nurse and physician available in the ED during a period of 15 minutes around each time point. The researcher in place (JW) ensured that a minimum of one nurse and physician per staffed area in the ED completed the assessment. During the validation at Malmö, assessments were collected from nurses and doctors via a computerized questionnaire without a researcher present.

### Statistical Analysis

All statistical analyses were performed using the IBM SPSS version 21 (IBM Corporation, NY, US). Pearson correlation, T-Test for mean value comparison and Fisher’s exact test were used to analyze the results. A result was classified as statistically significant if the p-value was less than 0.05 and the 95% confidence interval did not include zero.

### Ethics

The present study was carried out in accordance with The Declaration of Helsinki [10] and was a quality assessment initiative that included no single patients identifiable to the researchers. The personnel participated as part of their normal duty, and the data on their assessments and performance were collected and analyzed anonymously. Written information about the study was sent by email to the staff at each ED prior to the study and was also present together with the assessment forms. Participating nurses or physicians were able to opt out at any time, and consented to participation by making the assessments. This type of quality assessment study is exempt from review by the regional ethics committees in Sweden.

## Results

### Workload Assessments

Assessments were collected at 50 time points at each ED, and out of a total of 250 time points, 197 (78.8%) complete and 36 (14.4%) partially complete workload assessments were obtained. The fraction of complete assessments were equally distributed Monday through Sunday as well as over the different time points of the day. Nurses assessed the workload at more time points than physicians (89.2% vs. 82.8%, p = 0.053). Both nurse and physician assessment scores were normally distributed. The correlation between the nurse and physician scores was r^2^ = 0.407 (p < 0.0001). Physicians assessed the workload as somewhat higher than nurses, with average scores of 3.32 and 3.19 respectively (p = 0.75).

### Regression Model

**Fig 1:**
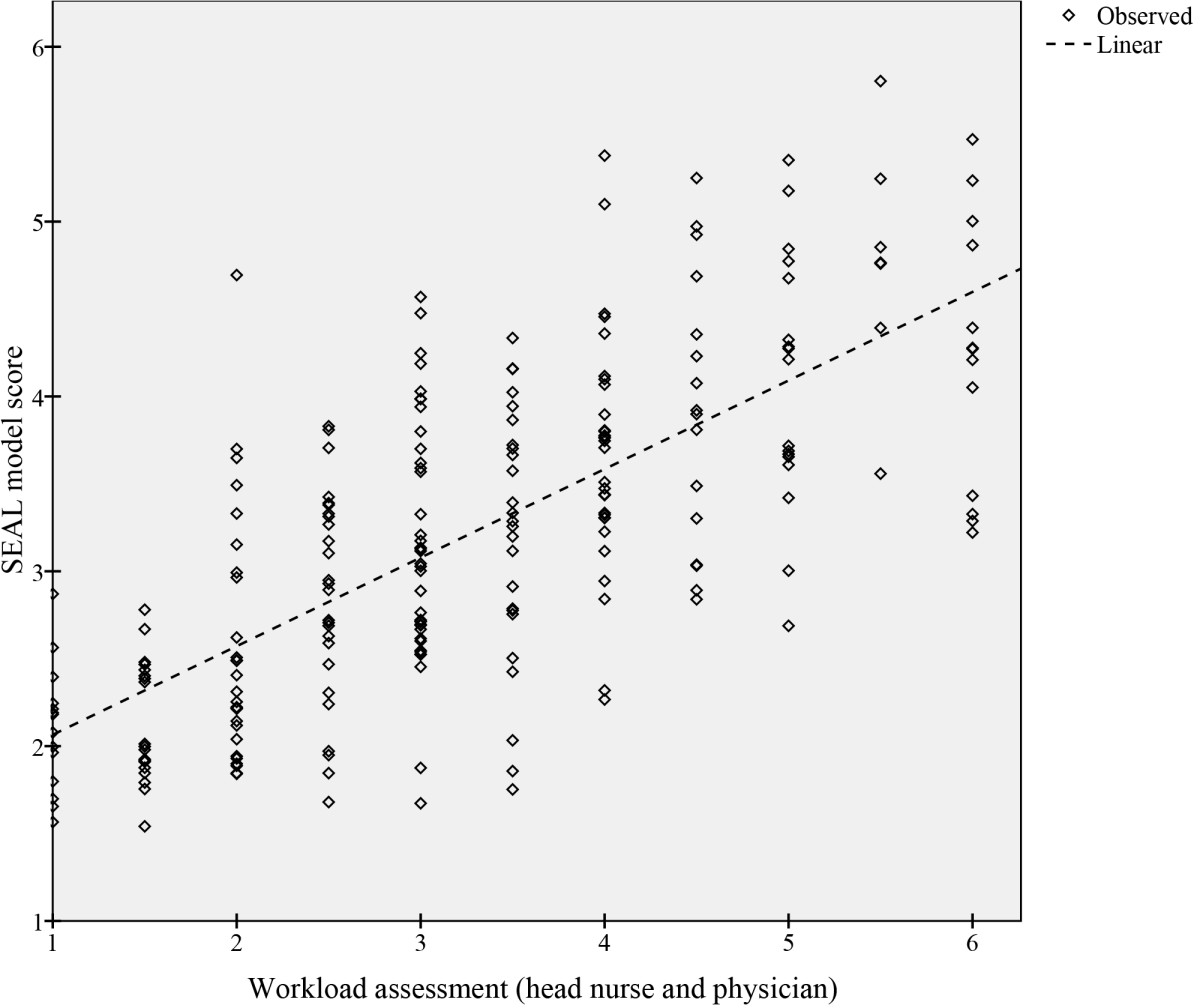
Correlation between the SEAL model score and workload assessments. Variables in bold are included in the final model.

Four of the 14 variables: *Patient hours*, *High priority*, *Awaiting MD* and *Occupancy* each demonstrated a significant correlation to the assessed workload (Table 2). When analyzed together in a reduced model, these four variables explained 96.4% of the full model based on the r² value, and correlated well with the workload assessments (r² = 0.509, p < 0.001, Fig 1). The correlation between the assessments and *Occupancy* alone was r^2^ = 0.334, p < 0.001. A retrospective calculation of the reduced model score (1–6) over the initial collection period (503 h) gave an average score of 3.5, 3.4, 3.3, 2.9 and 3.0 for EDs A-E respectively. The difference in mean score between the validation EDs, Lund (A) and Malmö (B), was not statistically significant (p = 0.084). The SEAL score is calculated by adding 1.589 to the sum of the four variables highlighted (bold) in Table 2, each variable multiplied by its coefficient. Its value varies between 1 and 6, 6 representing the highest workload.

**Table 2:** List of analyzed variables.

Variable	p-value	coeffcient	95% CI	Measure
Priority	0.23	-0.64	-0.79–0.19	Average priority for all patients in the ED
Triage priority	0.48	-0.40	-0.58–0.27	Average initial priority as assessed in triage for all patients in the ED
**High priority**	**0.01**	**1.80**	**0.39–3.21**	**Ratio: number of high priority patients (1 and 2) by number of patients in the ED**
**Awaiting MD**	**<0.01**	**1.39**	**0.58–2.21**	**Ratio: total time patients spent waiting for a doctor by number of patients in the ED**
Average time	0.13	0.07	-0.02–0.14	Average time in hours spent in the ED discharged patients
Longest stay	0.79	0.02	-0.03–0.04	Longest stay for any patient in the ED
**Patient hours**	**<0.01**	**14.73**	**11.12–18.34**	**Ratio: total time, in hours, spent by all patients in the ED by average daily visits**
**Occupancy**	**<0.01**	**-1.10**	**-1.79–-0.41**	**Ratio: number of patients in the ED by number of ED beds**
Occupancy rate	0.72	0.03	-1.01–1.58	Ratio: number of registered patients by number of ED beds
Average volume	0.79	-0.12	-12.4–9.48	Ratio: number of patients by the average daily visits
Admit index	0.95	0.00	-26.3–28.2	Ratio: number of patients waiting for admission by number of hospital beds
Unseen	0.81	-0.03	-1.50–-1.72	Ratio: unseen patients by number of ED beds
MDs	0.43	-0.04	-1.92–0.83	Ratio: number of MDs by number of patients
Nurses	0.38	-0.05	-1.84–0.70	Ratio: number for nurses by number of patients

Variables included in the reduced model in bold. All variables (numerators in ratios) were measured during the hour prior to the assessments

### Validation

Workload assessments at Lund were collected from 219 nurses and 174 physicians at 91% (n = 32) of the predefined time points, and at Malmö from 307 nurses and 195 physicians at 70% (n = 49) of the time points. On average, 5.9 nurses and 5.1 physicians at Lund, and 6.5 nurses and 4.1 physicians at Malmö, assessed the workload at each time point. The calculated model score correlated well with the workload assessments at both EDs as shown in Table 3. The assessments made by the head nurse and physician were noted and compared with the mean assessments of the rest of the staff at 28 time points (80%) at Lund, and at 36 time points (51%) at Malmö. Correlations between assessments made by the head staff and the rest of the staff were poor at Lund (r^2^ = 0.160, p = 0.035) and moderate at Malmö (r^2^ = 0.359, p < 0.001).

**Table 3:** Pearson correlations between the reduced model and assessment data sets.

		*r* ^*2*^	p	n
**Primary data set**	Full model (14 variables)	0.964	< 0.001	250
	Head Staff	0.509	< 0.001	233
**Validation Lund**	All Staff	0.641	< 0.001	32
**Validation Malmö**	All Staff	0.624	< 0.001	49

## Discussion

This study shows that it is possible to objectively quantify the degree of crowding based on workload in Swedish EDs using easily accessible data from the patient log system. Using a multiple linear regression analysis we created a model, independent of ED size, which performed well when validated against new data from two of the EDs. If able to identify times of crowding our model has the potential to help evaluate interventions to decrease crowding in the ED, as well as to help comparing different EDs.

To make our model suitable for general use and potentially for benchmarking, we chose variables that are independent of ED size and that are based on data which should be available in any ED. Our reduced and final model consisted of four variables (*High priority*, *Awaiting MD*, *Patient hours*, *Occupancy*) out of an initial 14. The model thus partly covers two out of three areas in the conceptual input-throughput-output model of ED crowding described by Asplin et al [11]. The input factor, the inflow of patients, is represented by *High priority*, *Patient hours* and *Occupancy* and it is reasonable to assume that the inflow of patients has a significant impact on the staff’s workload and the level of crowding. *Awaiting MD*, *Patient hours* and *Occupancy* can be viewed as measures of throughput. *Patient hours* and *Occupancy* both reflect aspects of the number of patients present in the ED and *Patient hours* was positively correlated with staff workload, whereas *Occupancy*, perhaps surprisingly, was found to be negatively correlated with workload. Our interpretation of this is that *Occupancy* and *Patient hours* are linked, and that higher *Occupancy* at unchanged *Patient hours* means quicker (and perhaps less complicated) management of each patient, which tends to decrease workload. Similarly, high throughput yields shorter visits, and thus fewer *Patient hours* with relatively preserved *Occupancy* and a lower model score. The extent of “boarding” patients, represented by *Admit index* in our study, has been shown internationally to correlate with ED crowding [2, 12], but in the present study the *Admit index* did not correlate significantly with assessed workload. It seems reasonable to assume that the problem of “boarding”, and its impact on the staff workload, is of different magnitude in different healthcare systems.

Our model correlated well with the assessed workload in the validation data sets, and the results are comparable with those described for NEDOCS [6]. A perfect correlation would be very hard to achieve for a variable as complex as workload, which encompass all aspects of the ED work and varies among individuals. Improved correlation might increase the accuracy of the model but more important is whether the model can identify situations when crowding affects patient care and outcomes, and discriminate well between different levels of crowding. We believe this should be the primary objective for further studies. The correlation between our model and the validation datasets was better compared to the initial dataset (Table 3), and this could in part be explained by the fact that we asked all staff during the validation, but only the head staff during the primary data collection. It seems likely that averaged workload assessments from the entire staff is less variable than data from the head staff only. Indeed, during the validation the workload assessments by the head staff and all staff were not highly correlated.

Both NEDOCS and EDWIN were derived in EDs with perceived high prevalence of crowding, which may explain their less than perfect validation results in EDs with lower crowding prevalence [6]. We addressed this issue by deriving our model in five EDs of different sizes and with presumable differences in crowding prevalence. We then validated our model in the two EDs with the largest census, and the highest average workload scores during the study period. We believe that this increased the reliability of the validation results, and that they indicate that the model is able to estimate crowding based on workload in Swedish EDs. Before general use however, the model should be validated at multiple EDs outside Region Skåne, preferably involving the entire staff of physicians and nurses.

Although the term crowding is well established in the literature, there is no clear definition of it. The NEDOCS model was developed based on staff assessments of the level of crowding, as well as on physicians’ feelings of being rushed. Other studies in the United States have used “ambulance diversion” as the reference standard for crowding [6]. *Occupancy*, the ratio of ED beds to patients, have been used as a single marker for ED crowding [13]. However, *Occupancy* mainly reflects the physical resources (beds, rooms) of the ED and not the human resources (staff) which we believe are equally important for optimal ED function. In the present study, we based our model on workload since it encompasses both the human and physical resources at the ED, and since it is reasonable to assume that most of the negative effects of crowding on care quality [5] are mediated via a high staff workload. Further, the term crowding is not commonly used in Swedish EDs, whereas workload is a familiar term for the staff and administrators. Since the main concern with crowding is its tendency to decrease the quality of care [5], we believe that a definition should ideally be based on measures of care quality. This also implies that crowding models should in the end be validated directly against quality of care (e.g. patient outcome) and not only against the staffs’ assessment of the workload or the crowding. Our present results may thus be a stepping-stone towards further research. As mentioned, we based our analysis on workload since it might be more directly related to the quality of care than crowding per se. At least to the layperson, the term crowding implies that a large number of patients are present at the ED, but it says little about how sick these patients are, or how the personnel handles the situation. Numerous low acuity patients in the ED may not necessarily decrease the quality of care, whereas a few very sick patients may severely compromise the care given at the entire ED. The term crowding is appealing since it is “visual” and easily understandable to the public, but we propose that it could be supplemented (or at times perhaps even replaced) by “ED patient load” or “staff workload”, which may better describe the actual problem at hand.

International research has described the problems of ED crowding and is now beginning to focus on solutions. In many countries however, at least in Sweden, there is a need for more information about the scale of the problem and its underlying causes. Our model has the potential to provide such information. Further, large resources are used in numerous local initiatives to improve the quality of ED care. These initiatives need to be based on reliable data on the operation of the single ED and, ideally, of other similar EDs. The model presented here will allow automatic (computerized), continuous, real-time or retrospective monitoring of the workload at the individual ED, and might also be used to compare the operation of different EDs. In Sweden, the developing national quality registry ANSWER [14] might be of help in this respect.

## Limitations

Our model correlated well with staff assessments of workload, but additional information is needed on how to interpret the model score. The test characteristics and cut off values for crowding have not been fully explored in this derivation project. Further studies will focus on analyzing the correlation between the score and quality of care.

Many factors affecting the workload are not stored in the patient log system used, and were hence not possible to include in the model. Dissatisfied and angry patients, novice personnel or computer problems are all factors that may affect actual or perceived workload but are not available in the system. It is of course possible that our model can be refined and optimized with the addition of more variables, to yield even better estimates of the ED workload.

Most variables in the patient log system are manually entered by the staff, but our reduced model primarily includes relatively dependable system variables such as patient numbers, occupancy and priority. However, Awaiting MD and some variables excluded in the reduced model depend more on correct registrations by the personnel, and the accuracy of these variables might have varied in our material. Staff variables, MDs and Nurses, based on assigned nurses and physicians in the log system are likely more accurate at smaller EDs where fewer personnel have administrative or supporting work roles.

Our model was based on the use of the RETTS [10] system and hence our model might not be valid in EDs using other triage or acuity systems. In Sweden however, a majority of EDs use the RETTS system, and the range in size of the EDs included in this study is representative of most EDs in Sweden [1]. Our model should therefore be applicable at a majority of the Swedish EDs.

## Conclusions

In this study, we present a model to estimate the level of crowding based on workload in Swedish EDs. The model may be applicable to EDs with different sizes and characteristics, and could be used for monitoring of ED workload retrospectively and in real time, before and after organizational changes, and for comparison with other EDs. Before widespread use however, the model should be validated at EDs outside Region Skåne, and ideally also tested against data on ED quality of care. We believe our model has the potential to help improve care for our patients as well as working conditions for the personnel.
